# Supplementary material for: COVID-19 and the Change in Lifestyle: Bodyweight, Time Allocation, and Food Choices
Source: Int J Environ Res Public Health. 2021 Oct 8;18(19):10552. doi: 10.3390/ijerph181910552 (PMC8508365; doi:10.3390/ijerph181910552)
Supplement: Supplementary file 1 [file ijerph-18-10552-s001.zip › ijerph-1378882-supplementary.pdf]

Supplementary Materials

Table S1. VIF value for Bodyweights and Time Allocation

**Variable**	**Δ Weight t1**	**Δ Weight t2**	**Δ Exercise time t1**	**Δ Exercise time t2**	**Δ Entertainment time t1**	**Δ Entertainment time t2**
Risk aversion	1.05	1.05	1.06	1.05	1.06	1.06
Fear of resurgence	1.06	1.06	1.07	1.07	1.08	1.08
Size of social network	1.23	1.21	1.24	1.22	1.24	1.22
Confirmed case	4.85	5.47	4.86	5.48	4.88	5.48
Search frequency	2.45	2.53	2.46	2.53	2.46	2.54
Lockdown duration	1.11	1.10	1.45	1.46	1.45	1.47
Package delivery restriction			1.41	1.41	1.41	1.41
Duration of COVID					1.06	1.07
Experience starvation					1.11	1.12
Stores nearby			1.08	1.08	1.08	1.08
Women	1.09	1.09	1.09	1.09	1.10	1.09
Age	1.42	1.41	1.43	1.41	1.44	1.42
Married	1.55	1.55	1.56	1.55	1.56	1.55
Education	1.27	1.27	1.28	1.28	1.28	1.28
Health status	1.14	1.14	1.14	1.14	1.14	1.14
Income	1.26	1.26	1.26	1.26	1.28	1.28
Family size	1.39	1.40	1.40	1.40	1.40	1.40
Either child or elderly at home	1.61	1.61	1.62	1.62	1.62	1.62
Household member a medical staff	1.09	1.09	1.09	1.09	1.13	1.13

Table S2. VIF value for Food Choices

**Variable**	**Δ Online food Purchase t1**	**Δ Online food Purchase t2**	**Δ Snack Purchase t1**	**Δ Snack Purchase t2**
Risk aversion	1.05	1.05	1.05	1.05
Fear of resurgence	1.07	1.07	1.08	1.08
Size of social network	1.24	1.22	1.23	1.22
Confirmed case	4.89	5.50	4.87	5.47
Search frequency	2.46	2.53	2.46	2.53
Lockdown duration	1.45	1.45	1.46	1.47
Package delivery restriction	1.41	1.41	1.41	1.41
Duration of COVID			1.06	1.07
Diagnosed	1.15	1.16		
Δ Price			1.25	1.12
Women	1.09	1.09	1.09	1.09
Age	1.43	1.42	1.44	1.42
Married	1.55	1.55	1.56	1.55
Education	1.28	1.28	1.28	1.28
Health status	1.14	1.14	1.14	1.14
Income	1.26	1.26	1.26	1.26
Family size	1.40	1.40	1.41	1.40
Either child or elderly at home	1.62	1.62	1.62	1.62
Household member a medical staff	1.17	1.17	1.12	1.12
